# Modulation of FOXO3 Transcriptional Regulation by Cisplatin and Curcumin in Human Embryonic Kidney Cells

**DOI:** 10.3390/ijms27208970

**Published:** 2026-10-09

**Authors:** Edson Coronado-Moreno, Victor Mendoza-Santos, Isabel Larre, Diana González-Cázares, Elisa Dorantes, Oscar Medina-Contreras, Adriana Sánchez-Boiso, Benjamin A. Rodriguez-Espino, Omar Guadarrama, Mara Medeiros

**Affiliations:** 1 Facultad de Medicina, National Autonomous University of Mexico, Mexico City 04510, Mexico; edsonmoreno.05@gmail.com; 2 Unidad de Investigación y Diagnóstico en Nefrología y Metabolismo Mineral Óseo, Hospital Infantil de México Federico Gómez, Mexico City 06720, Mexico; vicco2912@nube.unadmexico.mx (V.M.-S.); isabelarrep@gmail.com (I.L.); dianatochi@gmail.com (D.G.-C.); adrianaboiso@gmail.com (A.S.-B.); rodriguezespinoba@gmail.com (B.A.R.-E.); eogd_83@hotmail.com (O.G.); 3 Biobanco de Células, Hospital Infantil de México Federico Gómez, Mexico City 06720, Mexico; elisadorantes@gmail.com; 4 Laboratorio de Inmunometabolismo, Hospital Infantil de México Federico Gómez, Mexico City 06720, Mexico; 5 Dirección de Investigación, Hospital Infantil de México Federico Gómez, Mexico City 06720, Mexico

**Keywords:** cisplatin, renal damage, FOXO3, curcumin, nephrotoxicity, epigenetics

## Abstract

Cisplatin is a highly effective anti-neoplastic agent, but its clinical application is limited by its association with severe nephrotoxicity. Although extensive research has identified several mechanisms underlying cisplatin-induced nephrotoxicity, the precise molecular pathways remain incompletely understood. Recent studies have highlighted the role of intrinsic survival, senescence, and longevity signaling pathways, including those governed by the FOXO3 transcription factor. In this study, we examined the effects of cisplatin and curcumin on the DNA methylation status and mRNA expression of FOXO3 in human embryonic kidney (HEK293) cells. Our results indicate that the FOXO3 promoter is methylated in cisplatin-treated cells, and that cisplatin regulates FOXO3 transcription in a dose-dependent manner. Quantitative RT-qPCR revealed that moderate cytotoxic concentrations of cisplatin (10 and 40 µM) significantly downregulate FOXO3 mRNA. Furthermore, co-treatment with the antioxidant curcumin successfully restored and enhanced FOXO3 expression at moderate cisplatin doses (10 µM) but failed to rescue expression at high doses (40 µM). Moreover, curcumin treatment (10 µM) reverses the effects of low-dose, but not high-dose, cisplatin (40 and 60 µM). Taken together, these findings provide a foundation for understanding the epigenetic and transcriptional regulation of FOXO3 and highlight its potential as a target for antioxidant-mediated nephroprotection and its role in cell survival.

## 1. Introduction

Cisplatin (cis-diamminedichloroplatinum(II)) is a highly effective platinum-based chemotherapeutic agent that has been used for over 50 years to treat various solid tumors, including head and neck, bladder, testicular, ovarian, uterine, cervical, and breast cancers, significantly extending patient survival [1,2,3]. As an alkylating agent, its square-planar chemical structure consists of a central platinum atom bonded to two chloride ions and two inert ammonia ligands in a *cis* configuration. Cisplatin binds primarily to guanine and adenine bases on DNA to form 1,2-intrastrand cross-links, blocking replication and transcription in cancer cells.

Despite its efficacy, treatment with cisplatin is frequently complicated by nephrotoxicity, which presents as acute kidney injury in approximately 25–35% of patients in a dose-dependent manner [4]. Current preventive strategies, such as aggressive hydration and magnesium supplementation, only partially alleviate this toxicity because they do not target the underlying molecular mechanisms [5]. At the cellular level, cisplatin accumulates in the proximal tubules at concentrations five times higher than in the blood [6]. This triggers a cascade of cellular stress responses, including DNA damage, p53-dependent apoptosis, oxidative stress, endoplasmic reticulum stress, and mitochondrial dysfunction. While cell death mechanisms such as apoptosis, necrosis, necroptosis, and ferroptosis have been extensively explored in this context, recent studies increasingly highlight the role of intrinsic survival signaling and epigenetic regulators such as Sirt1 and FOXO in propagating tubular cellular damage [7,8,9,10,11].

The Forkhead box class O (FOXO) transcription factors represent a subfamily of the FOX superfamily, which in mammals consists of FOXO1, FOXO3, FOXO4, and FOXO6. These proteins play pivotal roles in diverse physiological and pathological processes, including cell cycle progression, glucose homeostasis, DNA repair, autophagy, apoptosis, and stress resistance [12]. Studies have identified FOXO3 as a key regulator of cell death triggered by chemotherapeutic agents across different cancers, demonstrating that the depletion of FOXO3 levels is often sufficient to abrogate drug-induced apoptosis; moreover, SIRT1-FOXO3-p53 is a signaling pathway active during cisplatin-dependent nephrotoxicity [11,13].

Curcumin—or 1,7-bis(4-hydroxy-3-methoxyphenyl)-1,6-heptadiene-3,5-dione (diferuloylmethane)—is a natural bioactive polyphenolic compound that mediates a wide spectrum of biological functions. In current research, curcumin is considered an agent with antioxidant, anticancer, anti-inflammatory, and potentially renoprotective properties [14].

In the context of the kidney, cisplatin-induced nephrotoxicity has been associated with the modulation of critical antioxidant transcription factors, including Nrf2 and FOXO3a, alongside SIRT1 [15]. It has been shown that the FOXO3 protein is regulated by cisplatin in vitro and in animal renal models of nephrotoxicity [16]; however, its epigenetic and transcriptional regulation by cisplatin remains poorly understood. To investigate the link between FOXO3 and cisplatin-induced nephrotoxicity, and to explore potential protective interventions using antioxidants such as curcumin, we evaluated the DNA methylation status and quantitative mRNA expression of FOXO3 in HEK293 cells following cisplatin exposure. It has been shown that curcumin regulates the activity of the FOXO3 protein through specific signaling pathways, including dephosphorylation, acetylation, and, more recently, transcriptional FOXO3 levels [11,17,18].

## 2. Results

### 2.1. Effect of Cisplatin on the Morphology and Viability of HEK293 Cells

**Figure 1:**
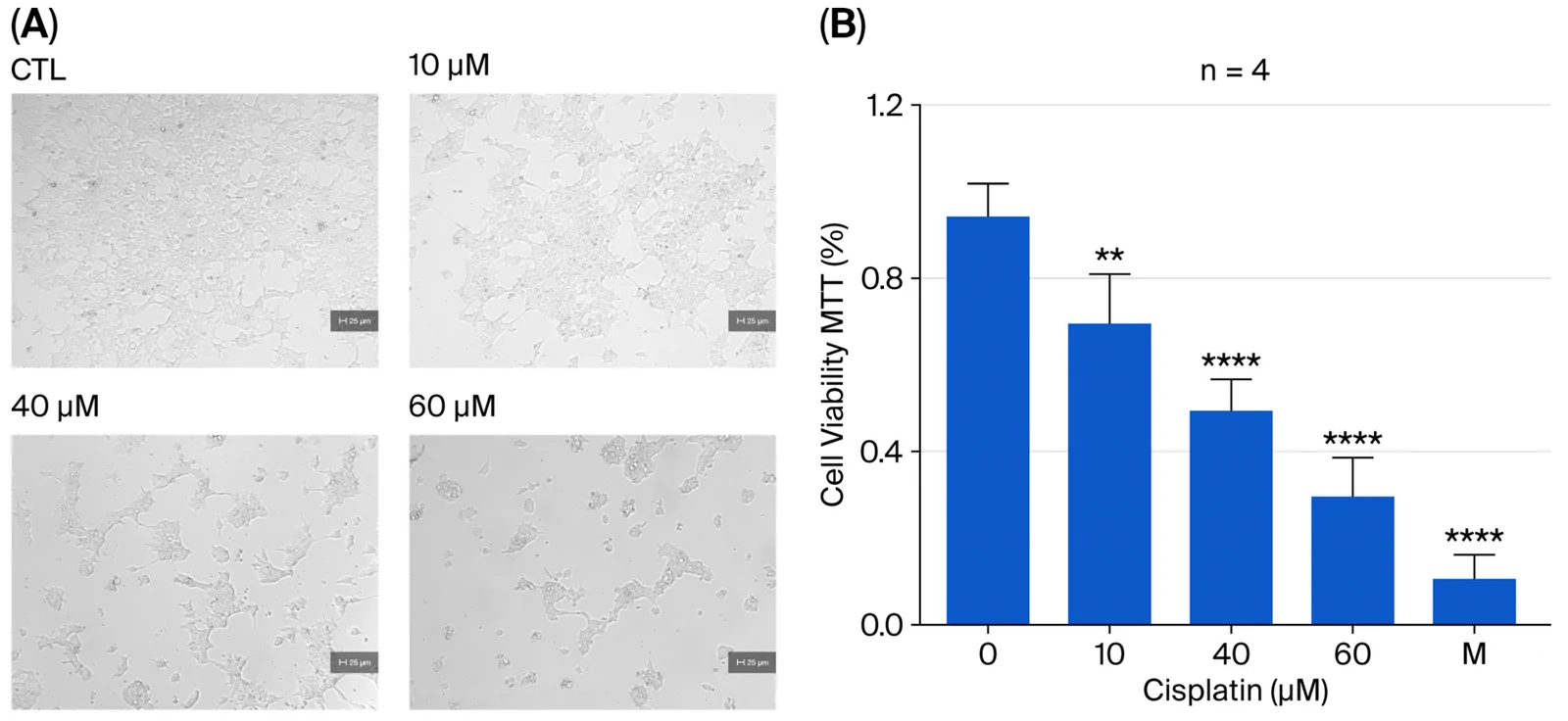
The effect of cisplatin on the morphology of HEK293 cells observed using optical microscopy and MTT assay. (**A**) Representative image of control monolayers (DMSO) and 10–60 μM of cisplatin at 48 h. (**B**) Statistical analysis of A. M corresponds to mitomycin 25 μM, a positive control. All scale bars are 25 µm; data are means ± SEM. (n = 3), ** *p* < 0.01, **** *p* < 0.0001 compared to vehicle control (DMSO).

Previous reports indicate that cisplatin induces time- and dose-dependent toxicity in renal epithelial cells [17,19]. To evaluate toxicity in our model, we analyzed the morphology and viability of HEK293 cells following 48 h exposure to varying concentrations of cisplatin (10 to 60 μM). Doses of up to 10 μM resulted in mild changes in viability, retaining approximately 70% viability with no severe morphological alterations (Figure 1A,B). However, cells treated with higher doses (40 μM and 60 µM) exhibited prominent morphological alterations, cellular detachment, and a significant decrease in cell viability, retaining only ~50% and ~30% viability, respectively, compared to the vehicle control (Figure 1B), similar to previous reports [17,19].

### 2.2. Effect of Cisplatin on FOXO3 Methylation

**Figure 2:**
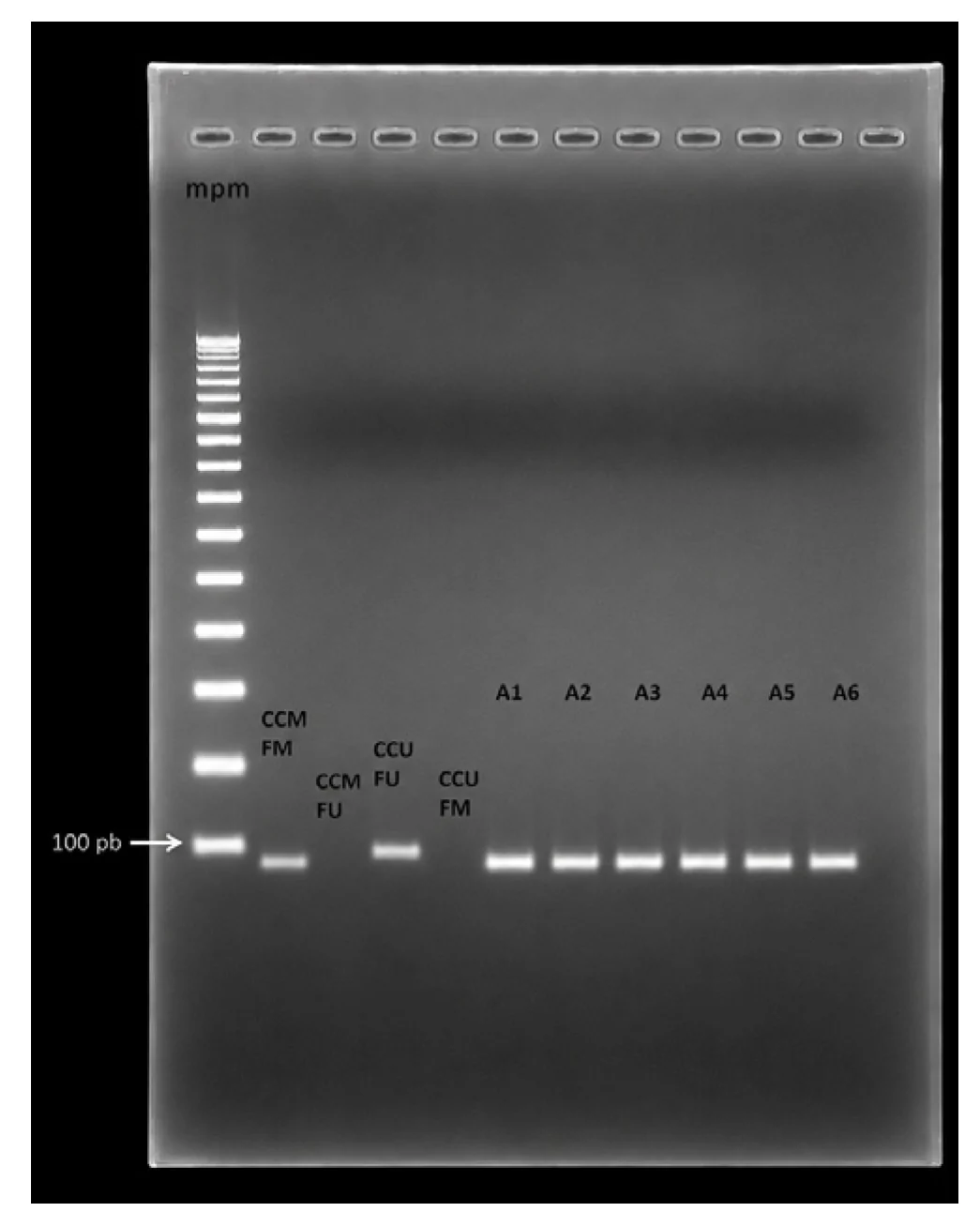
Analysis of the methylation status of the FOXO3 gene in HEK293 cells treated with different concentrations of cisplatin. A representative image of methylation-specific PCR (MSP) gel electrophoresis performed on bisulfite-treated genomic DNA is shown. The bands at the bottom correspond to detection of the methylated FOXO3 promoter. The image shows 2% agarose gel electrophoresis (stained with GelRed^®^) displaying the following: MPM = 100 bp marker; CCMFM = 71 bp amplicon (using commercial methylated control DNA and methylated FOXO3 primers); CCMFU = no amplicon observed (using commercial methylated control DNA and unmethylated primers); CCUFU = 81 bp amplicon (using commercial unmethylated control DNA and unmethylated FOXO3 primers); CCUFM = no amplicon observed (using unmethylated control DNA and primers for methylated FOXO3, A1–A6); and a 71 bp amplicon obtained from cultured cells exposed to varying concentrations of cisplatin for 48 h.

To evaluate whether cisplatin exposure induces qualitative epigenetic modifications, methylation-specific PCR (MSP) was performed. In the control monolayer (Figure 2, A1), a basal methylation signal was observed in the FOXO3 promoter. Similar qualitative methylation signals were maintained in cells treated with 2.5, 5, 10, 20, and 40 μM cisplatin (Figure 2, A2–A6). While the persistent presence of methylation was confirmed across all treatments, this traditional MSP approach highlights the need for future high-resolution quantitative assessments to determine whether subtle epigenetic shifts occur during cisplatin exposure.

### 2.3. Quantitative Analysis of FOXO3 mRNA Expression Using RT-qPCR

**Figure 3:**
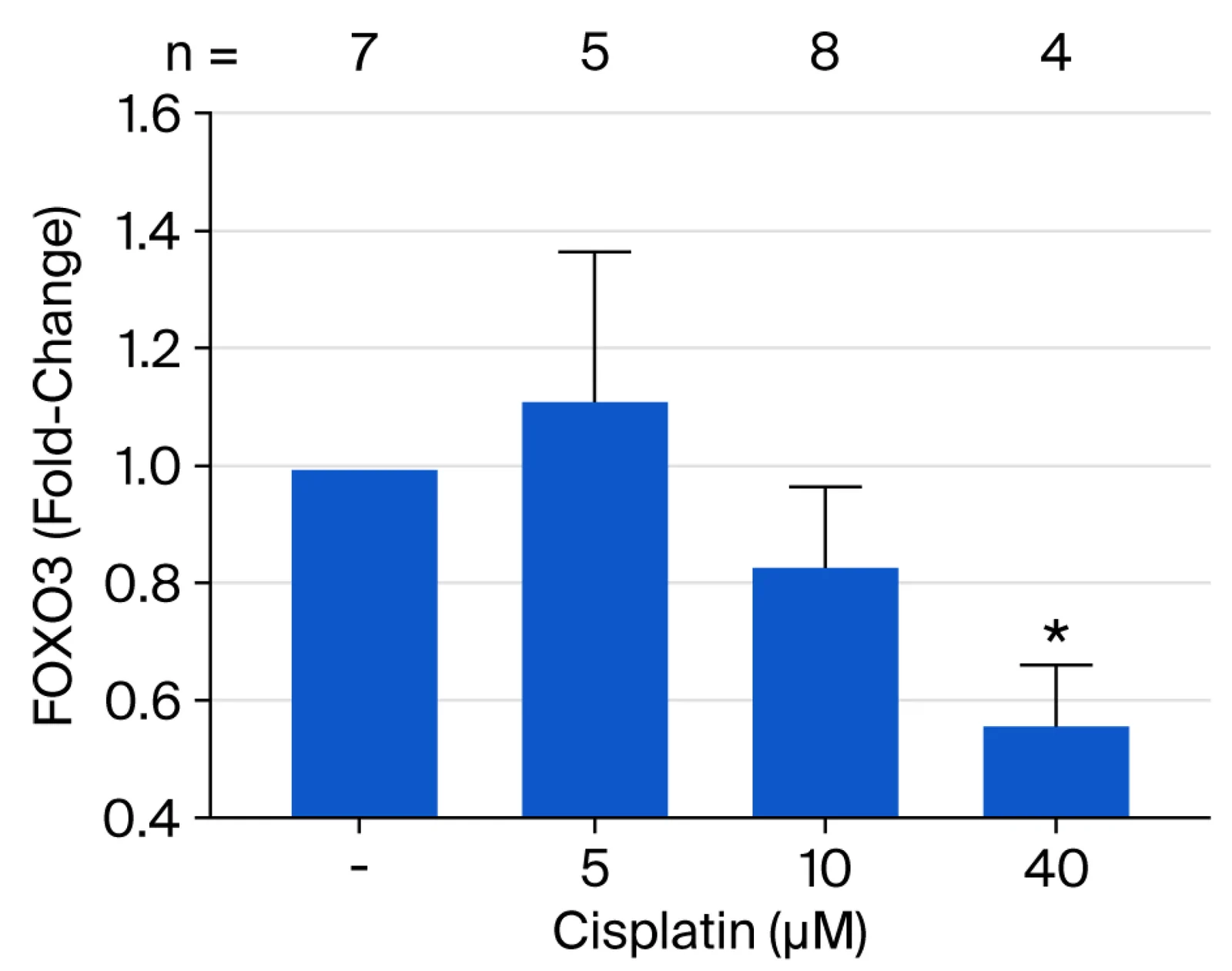
Dose-dependent modulation of FOXO3 mRNA by cisplatin. RT-qPCR analysis of FOXO3 mRNA expression in HEK293 cells treated with 0, 5, 10, and 40 μM cisplatin for 48 h. Values represent fold-change relative to control ± SEM (n = 8–4). * *p* < 0.05.

To accurately quantify transcriptional changes, we performed RT-qPCR following 48 h of cisplatin treatment. Transcriptional profiling revealed clear dose-dependent suppression in response to cellular stress (Figure 3). While a low dose of cisplatin (5 μM) induced a slight, non-significant increase in expression, moderate to highly cytotoxic concentrations (10 and 40 μM) significantly downregulated FOXO3 mRNA levels (by ∼0.83- and 0.56-fold relative to the control, respectively). These data suggest that as cisplatin concentrations reach cytotoxic thresholds, FOXO3 transcription is actively suppressed.

### 2.4. Curcumin Reverses Cisplatin-Induced FOXO3 Downregulation at Moderate Doses

**Figure 4:**
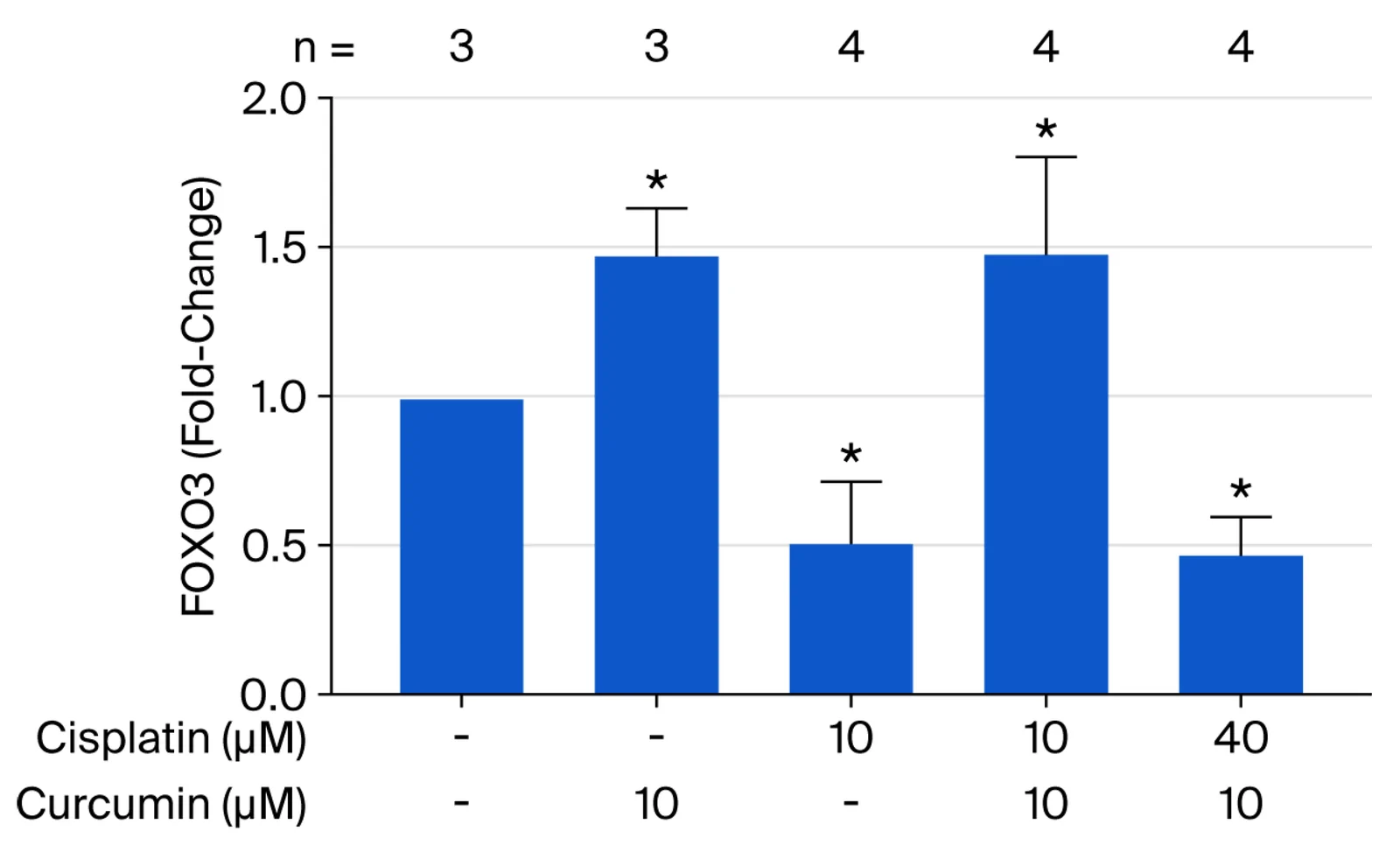
Curcumin rescues FOXO3 downregulation at moderate but not high cisplatin doses. RT-qPCR quantification of FOXO3 mRNA fold-change in cells treated with vehicle, curcumin alone (10 μM), cisplatin alone (10 μM), and co-treatments of cisplatin 10 μM and curcumin 10 μM (fourth column), and cisplatin 10 μM and curcumin 40 μM (last column). Values represent mean ± SEM (n = 3–4). * *p* < 0.05.

Because cisplatin at 10 μM and 40 μM suppressed FOXO3 transcription, we investigated whether the administration of the antioxidant curcumin could rescue its expression (Figure 4). Treatment with curcumin alone (10 μM) upregulated FOXO3 by ~0.5-fold compared to the control. Notably, co-treatment of cells with 10 μM cisplatin and 10 μM curcumin completely abolished cisplatin-induced FOXO3 downregulation, driving expression up to ~1.5-fold. However, when 10 μM curcumin was combined with a highly toxic 40 μM dose of cisplatin, this transcriptional rescue mechanism was overwhelmed, resulting in severe suppression of FOXO3 expression (~0.5-fold).

### 2.5. Effect of Co-Treatment on the Morphology and Viability of HEK293 Cells

**Figure 5:**
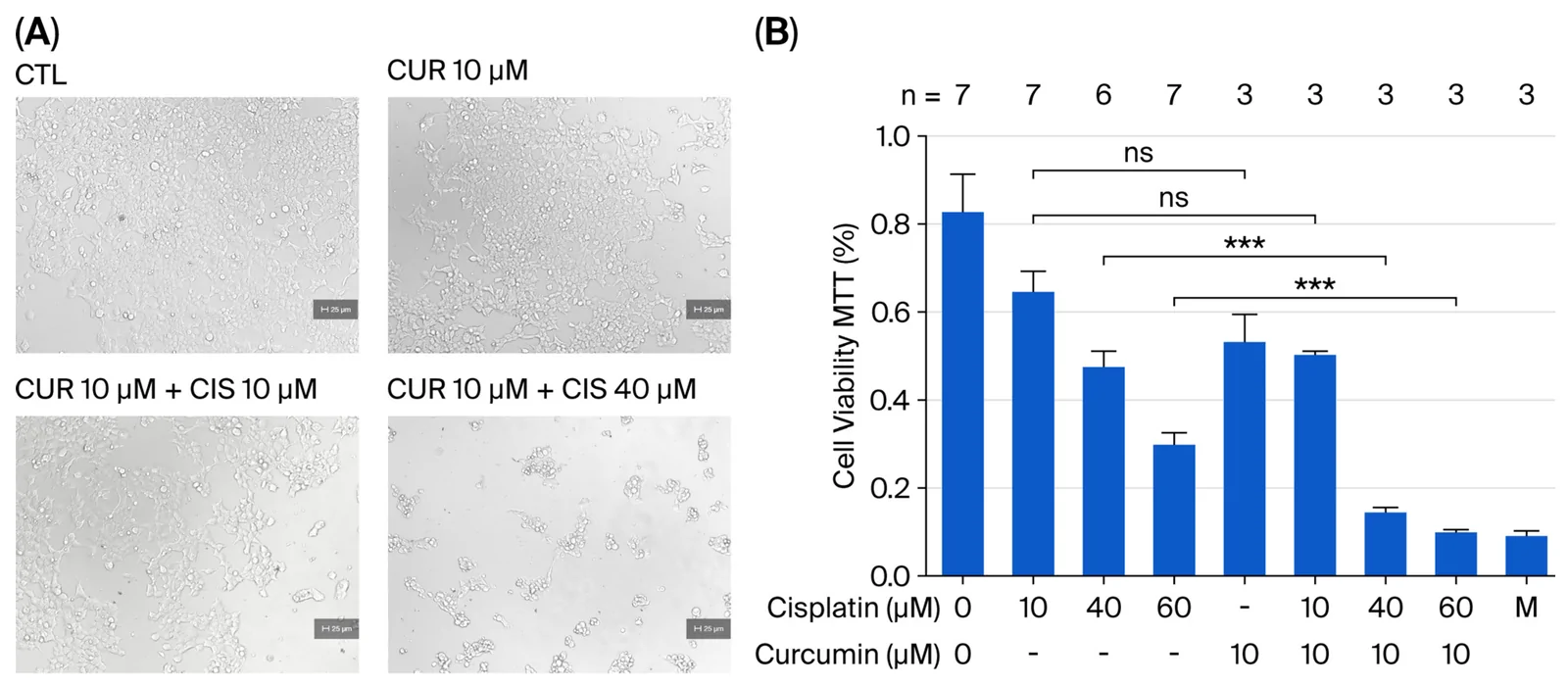
Curcumin modulates FOXO3 but not viability. (**A**) Images of control monolayers (DMSO), 10–60 μM of cisplatin, curcumin (cur 10 μM) and co-treatment of cisplatin and curcumin for 48 h (curcumin 10 μM + cisplatin 10, 40, or 60 μM). (**B**) Statistical analysis of cell viability via MTT assay with cisplatin, curcumin, and co-treatments (curcumin 10 μM + cisplatin 10, 40 or 60 μM). M corresponds to mitomycin 20 μM, a positive control. Data are means ± SEM (n = 7–3). All scale bars are 25 μm. Data are means ± SEM. ns = not significative, *** *p* < 0.001 compared to vehicle control (DMSO).

To evaluate whether the transcriptional rescue of FOXO3 by curcumin translates to cellular protection, we assessed cell viability using an MTT assay (Figure 5). In the control monolayer (DMSO), cells remained fully attached and viable. Viability with curcumin alone (10µM) did not differ from that with 10µM cisplatin. Interestingly, co-treatment with 10 µM curcumin and 10 µM cisplatin yielded no significant difference in viability compared to 10 µM cisplatin alone. Strikingly, at higher concentrations of cisplatin (40 µM and 60 µM), co-treatment with curcumin did not confer protection; rather, it significantly exacerbated cellular detachment and death compared to cisplatin alone (*p* < 0.001) (Figure 5B). Therefore, while curcumin modulates FOXO3 expression at moderate doses, it does not protect against cisplatin-induced cytotoxicity in this model and exacerbates toxicity at high concentrations.

## 3. Discussion

**Figure 6:**
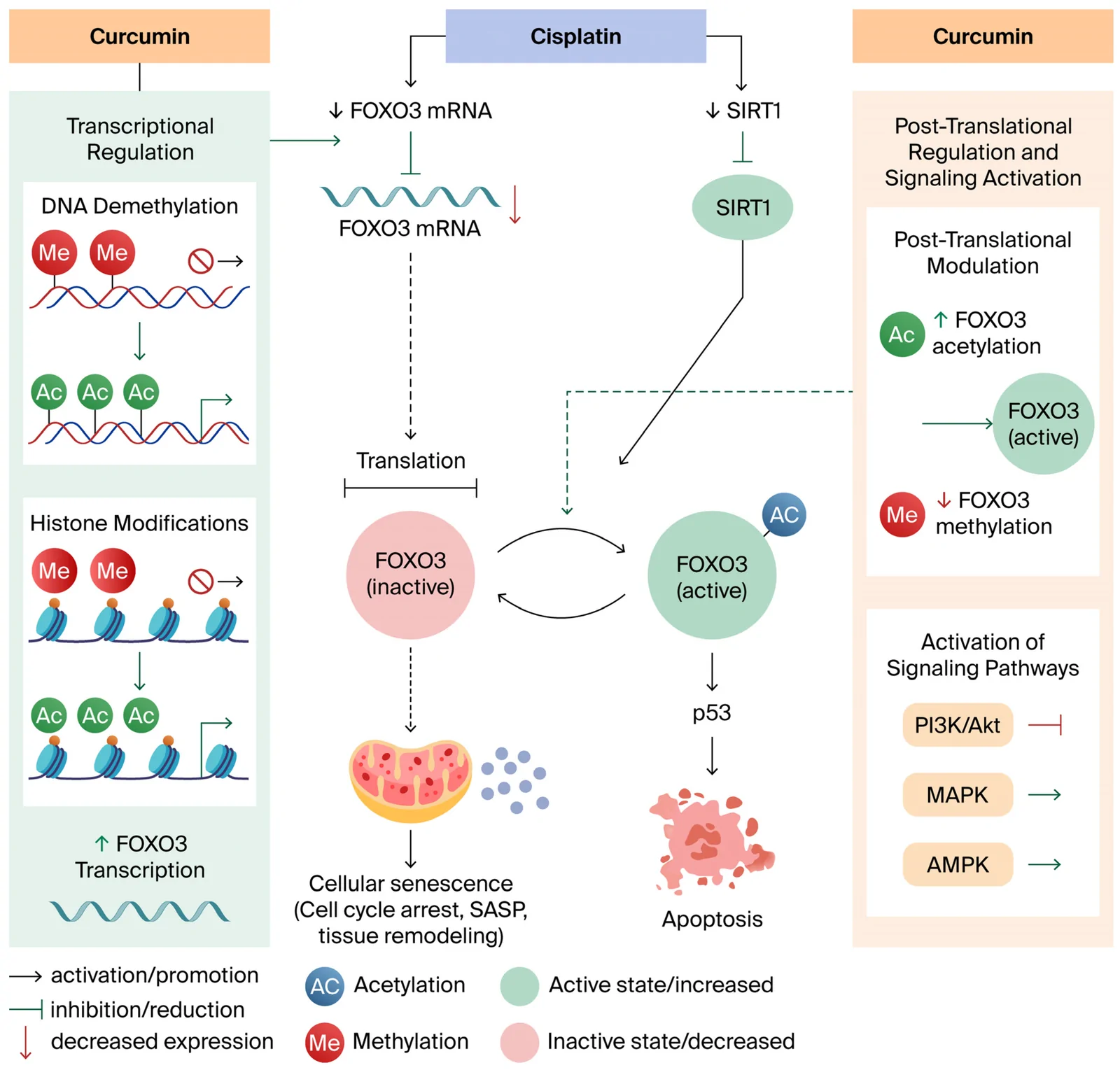
Schematic representation of mRNA regulation by cisplatin. FOXO3 mRNA levels decrease in the presence of cisplatin, which we hypothesize represents a compensatory response to limited activation of the FOXO3 protein through Sirt1/FOXO3-mediated apoptosis. Curcumin could regulate FOXO3 transcription in order to survive (co-treatment of curcumin and cisplatin 10 μM +10 μM).

Cisplatin is a highly effective chemotherapeutic agent, yet its clinical utility is frequently compromised by severe nephrotoxicity. Previous reports have demonstrated that cisplatin modulates the nuclear and cytoplasmic localization of FOXO3, as well as its post-translational modifications (e.g., acetylation) in renal cells [16]. While the canonical SIRT1/FOXO3/p53 signaling axis is known to regulate cisplatin-induced apoptosis (Figure 6) [20], the epigenetic and transcriptional regulation of FOXO3 during this stress response remains poorly understood. Our findings provide novel insights into this dynamic, demonstrating that cisplatin induces a complex, dose-dependent transcriptional suppression of FOXO3 in human embryonic kidney (HEK293) cells (Figure 6).

Epigenetically, our preliminary MSP results confirm that DNA methylation is constitutively present in the FOXO3 promoter of both untreated and cisplatin-treated cells. While this qualitative screening establishes a baseline epigenetic profile and shows that acute cisplatin exposure does not completely abolish or uniquely trigger promoter methylation, these findings warrant future high-resolution quantitative studies (such as qMSP or bisulfite sequencing) to precisely map localized and dynamic methylation shifts.

At the transcriptional level, our RT-qPCR data reveal that moderate to high injury-inducing concentrations of cisplatin (10 and 40 μM) progressively suppress FOXO3 mRNA expression. FOXO3 is a versatile transcription factor known to regulate both antioxidant defenses (such as SOD2 and catalase) and pro-apoptotic pathways. We hypothesize that this dose-dependent downregulation of FOXO3 may represent a compensatory cellular survival mechanism. By actively suppressing FOXO3 transcription during severe toxic stress, the cell may be attempting to limit the availability of the FOXO3 protein, thereby dampening the downstream activation of the SIRT1/FOXO3/p53 apoptotic cascade (Figure 6).

To test whether counteracting this suppression could mitigate toxicity, we utilized curcumin, a well-known polyphenol recognized for its potent antioxidant and nephroprotective properties [11,14]. Crucially, our study demonstrates that curcumin functionally modulates the FOXO3 pathway. Co-treatment with 10 µM curcumin successfully rescued and even enhanced FOXO3 expression in cells exposed to a moderate cisplatin dose (10 µM). However, at a higher cisplatin concentration (40 µM), the cellular defense machinery collapsed, leading to severe FOXO3 suppression despite the presence of curcumin [14,19]. Interestingly, while curcumin successfully rescued FOXO3 mRNA levels at 10 µM cisplatin, our MTT viability data revealed a distinct paradox: this transcriptional rescue did not translate into an improvement in cell survival. At 10 µM cisplatin/curcumin co-treatment, no significant difference in viability was observed. However, at high cisplatin concentrations (40 and 60 µM), curcumin co-treatment significantly exacerbated cytotoxicity. This divergence between mRNA rescue and cell viability suggests a complex dual role for FOXO3 in cell fate, either at a transcriptional or a functional level. If the cisplatin-induced suppression of FOXO3 is indeed a protective mechanism to avoid apoptosis, forcing FOXO3 upregulation with curcumin during cytotoxic stress might inadvertently prime the cell for death, explaining the heightened toxicity observed at higher doses. Alternatively, it is important to note that mRNA expression does not always strictly correlate with functional protein levels. FOXO3 activity is heavily governed by post-translational modifications (such as phosphorylation and acetylation) and intracellular shuttling. Therefore, restoring transcript levels alone may be insufficient to counteract cisplatin toxicity without appropriate downstream protein activation.

Finally, while our findings offer valuable molecular insights, the use of the HEK293 cell line represents a limitation of this study. Although HEK293 cells share numerous biochemical and molecular characteristics with early proximal tubule epithelial cells—making them a robust and reproducible in vitro model for preliminary mechanistic screenings—cisplatin-induced nephrotoxicity in vivo primarily targets the adult renal proximal tubule. Future investigations incorporating Western blot analysis, as well as validation in adult human proximal tubular cell lines (such as HK-2) or primary renal cells, will be essential to determine whether these transcriptional changes directly translate to altered FOXO3 protein levels and downstream apoptotic signaling.

## 4. Materials and Methods

### 4.1. Chemicals and Reagents

Cisplatin, curcumin, DMSO, and MTT were manufactured by Sigma-Aldrich (St. Louis, MO, USA). FOXO3 primers (Fw: 5′-TCTACGAGTGGATGGTGCGTTG-3′; Rv: 5′-CTCTTGCCAGTTCCCTCATTCTG-3′) and GAPDH primers (Fw: 5′-GGA TGA CCT TGC CCA CAG CCT-3′; Rv: 5′-CAT CTC TGC CCC CTC TGC TGA-3′) were purchased from T4 OLIGO (Guanajuato, Mexico).

### 4.2. Cell Culture and Treatment

The HEK293 cell line was purchased from the ATCC Cell Bank. Cells were cultured in Dulbecco’s modified Eagle’s medium (DMEM) supplemented with 10% fetal bovine serum (FBS) and 100 units/ml penicillin/streptomycin, in a humidified incubator with an atmosphere of 5% CO_2_ at 37 °C. All experiments were performed when cells reached 80% confluence. Cells were seeded in 6-well or 96-well plates and incubated overnight prior to treatments. Treatments included vehicle (DMSO), cisplatin (10 to 60 μM), and/or curcumin (10 μM) for 48 h.

### 4.3. DNA Extraction and Methylation-Specific PCR (MSP)

Following treatment, cells were harvested and lysed for genomic DNA extraction using the commercial Puregene DNA kit (Qiagen, Venlo, The Netherlands), according to the manufacturer’s protocol. Quantification was performed via spectrophotometry using a NanoDrop 2000 (Thermo Scientific, Waltham, MA, USA). DNA purity was assessed based on the A260/A280 ratio, with values between 1.8 and 2.0 considered indicative of high-quality DNA. DNA integrity was further verified by electrophoresis on a 2% agarose gel in TAE buffer and visualized using the intercalating dye GelRed (Biotium^®^, Fremont, CA, USA) under UV light.

To assess DNA methylation status, sodium bisulfite conversion was performed using the EZ DNA Methylation kit (Zymo Research, Tustin, CA, USA). Methylation-specific PCR (MSP) was performed using oligonucleotides designed with the MethPrimer program (identification sequence FOXO3cg06967118/FOXO3UP_UTR5). A standard methylated and non-methylated human DNA set (Zymo Research^®^) was used as the control. Amplification was conducted using the GoTaq^®^ Green Master Mix kit (Promega^®^, Fitchburg, WI, USA). Amplicons were visualized via 2% agarose gel electrophoresis (stained with GelRed^®^, Biotium, Inc., Fremont, CA, USA) against a 100 bp molecular weight marker to distinguish between methylated (M) and unmethylated (U) regions of the FOXO3 gene promoter.

### 4.4. Total RNA Extraction and cDNA Synthesis

Total RNA was extracted from HEK293 cells using TRIzol Reagent following the manufacturer’s recommended protocol. Cells were lysed by adding 1 mL of TRIzol per sample and homogenized to completely dissociate nucleoprotein complexes. Chloroform was added, and samples were shaken vigorously for 15 s, incubated for 3 min at room temperature, and centrifuged to separate the phases. The upper aqueous phase was recovered, and RNA was precipitated with isopropanol via gentle mixing and a 10 min incubation at room temperature. After centrifugation, the RNA pellet was washed with ethanol, centrifuged at 7500 rpm for 5 min at 4 °C, and air-dried at room temperature for 10 min. Finally, RNA was resuspended in RNase-free water and incubated at 55–60 °C for 10 min. RNA concentration and purity were determined using a NanoDrop 2000. cDNA synthesis was performed using total RNA and the High-Capacity RNA-to-cDNA™ Kit (Thermo Fisher Scientific, Waltham, MA, USA, Cat. No. 4387406). The resulting cDNA was quantified, yielding concentrations between 2203.7 and 2484.5 ng/μL with A260/A280 ratios of 1.85–1.89.

### 4.5. Quantitative Real-Time PCR (qRT-PCR)

Quantification of FOXO3 mRNA and the GAPDH reference gene was performed via quantitative real-time PCR using the Agilent AriaMx Real-Time PCR system (Agilent Technologies, Santa Clara, CA, USA) and the Brilliant III Ultra-Fast SYBR Green QPCR Master Mix kit (Agilent Technologies). Each reaction was prepared in a final volume of 10 μL and contained 2× Master Mix, 10 μM forward primer, 10 μM reverse primer, cDNA, and nuclease-free water. The thermal cycling program consisted of an initial DNA polymerase activation at 95 °C for 3 min, followed by 40 cycles of denaturation at 95 °C for 5 s and annealing/extension at 60 °C for 10 s, with fluorescence acquisition in the SYBR channel. Relative mRNA expression was calculated using the 2^−ΔΔ*Ct*^ method, normalized to GAPDH.

### 4.6. MTT Viability Assay and Microscopy

HEK293 cells were plated in 96-well plates and incubated overnight prior to treatment. Following a 48 h exposure to vehicle (DMSO) or cisplatin, 2 mg/mL MTT reagent (Sigma-Aldrich) was added to the DMEM, and the cells were incubated for an additional 2 h at 37 °C. The MTT reagent was then removed, the formazan crystals were dissolved in DMSO, and cell viability was analyzed by measuring absorbance at 550 nm using a SpectroStarNano microplate reader (BMG LabTech, Ortenberg, Germany). Morphological changes were documented via microscopy using a Leica DMi8 model.

### 4.7. Statistical Analysis

All data are expressed as means ± SEM derived from at least three separate experiments. Statistical analysis was performed using analysis of variance (ANOVA) followed by Bonferroni post hoc tests using GraphPad Prism software (v. 6.0.4). *p*-values of less than 0.05 were considered significant. Image analysis was performed using the ImageJ program (version 1.54p).

## 5. Conclusions

Cisplatin induces complex, dose-dependent transcriptional suppression of FOXO3 in human embryonic kidney cells. While antioxidant intervention with curcumin successfully rescues FOXO3 mRNA expression at moderate cisplatin doses, this transcriptional rescue does not correlate with improved cellular survival, and this combination at high doses exacerbates toxicity.

These findings suggest that FOXO3 suppression may act as a compensatory survival mechanism during cisplatin-induced stress. Future high-resolution epigenetic and translational studies focusing on the canonical SIRT1/FOXO3/p53 pathway are necessary to fully elucidate the pathophysiological impact of FOXO3 regulation and refine its potential as a therapeutic target in nephrotoxicity.

## Data Availability

The original contributions presented in this study are included in the article. Further inquiries can be directed to the corresponding author.

## Abbreviations

The following abbreviations are used in this manuscript.

AKI	Acute Kidney Injury
ANOVA	Analysis of Variance
ATCC	American Type Culture Collection
bp	Base Pairs
cDNA	Complementary Deoxyribonucleic Acid
DMEM	Dulbecco’s Modified Eagle’s Medium
DMSO	Dimethyl Sulfoxide
DNA	Deoxyribonucleic Acid
ER	Endoplasmic Reticulum
FBS	Fetal Bovine Serum
FOXO	Forkhead Box Class O
FOXO3	Forkhead Box O3
Fw	Forward (primer)
GAPDH	Glyceraldehyde 3-Phosphate Dehydrogenase
HEK293	Human Embryonic Kidney 293 (cells)
MSP	Methylation-Specific Polymerase Chain Reaction
MTT	3-(4,5-Dimethylthiazol-2-yl)-2,5-Diphenyltetrazolium Bromide
Nrf2	Nuclear Factor Erythroid 2-Related Factor 2
qMSP	Quantitative Methylation-Specific Polymerase Chain Reaction
RNA	Ribonucleic Acid
ROS	Reactive Oxygen Species
RT-qPCR	Quantitative Real-Time Polymerase Chain Reaction
Rv	Reverse (primer)
SEM	Standard Error of the Mean
SIRT1	Sirtuin 1
SOD2	Superoxide Dismutase 2
TAE	Tris-Acetate-EDTA
UNAM	Universidad Nacional Autónoma de México
UV	Ultraviolet
